# Supplementary material for: The Rice Serine/Arginine Splicing Factor RS33 Regulates Pre-mRNA Splicing during Abiotic Stress Responses
Source: Cells. 2022 May 30;11(11):1796. doi: 10.3390/cells11111796 (PMC9180459; doi:10.3390/cells11111796)
Supplement: Supplementary file 1 [file cells-11-01796-s001.zip › RS33_SI_18March2022.pdf]

SUPPORTING INFORMATION

The rice serine/arginine splicing factor RS33 regulates pre-mRNA splicing during abiotic stress responses.

Haroon Butt[1], Jeremie Bazin[2], Kasavajhala V.S.K. Prasad[3], Nour El-Islam[4], Martin Crespi[2], Anireddy S.N. Reddy[3], and Magdy M. Mahfouz[1,*]

[1]*Laboratory for Genome Engineering and Synthetic Biology, Division of Biological Sciences, 4700 King Abdullah University of Science and Technology, Thuwal 23955-6900, Saudi Arabia.*

[2]*CNRS, INRA, Institute of Plant Sciences Paris-Saclay IPS2, Univ Paris Sud, Univ Evry, Univ Paris-Diderot, Sorbonne Paris-Cite, Universite Paris-Saclay, Orsay, France.*

[3]*Department of Biology, Program in Molecular Plant Biology, Program in Cell and Molecular Biology, Colorado State University, Fort Collins, CO, USA*

[4]*Helmy Institute of Biomedical Science, Zewail City of Science and Technology, Ahmed Zewail Road, Giza, Egypt*

*Correspondence: Magdy M. Mahfouz (magdy.mahfouz@kaust.edu.sa)

Keywords

Pre-mRNA Splicing, Alternative Splicing, SR Proteins, Genome Engineering, abiotic stress

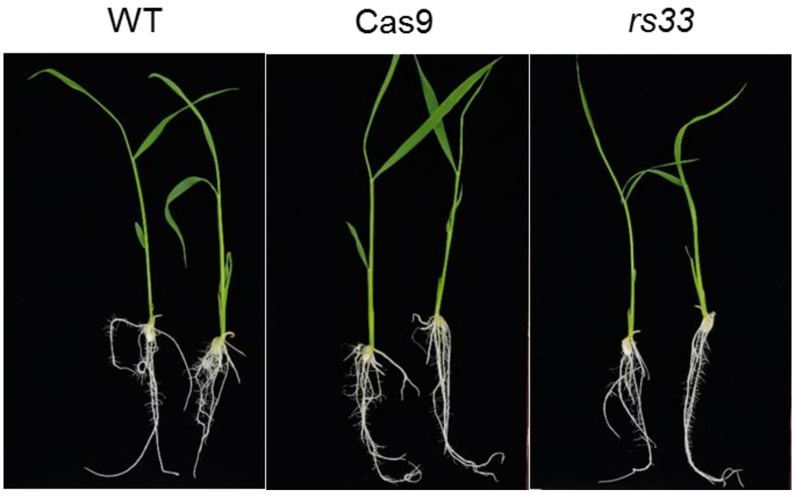

Figure S1: The *rs33* mutant phenotype

The WT, *Cas9*, and *rs33* seeds were germinated for ten days at the control temperature of 28ºC/26ºC.

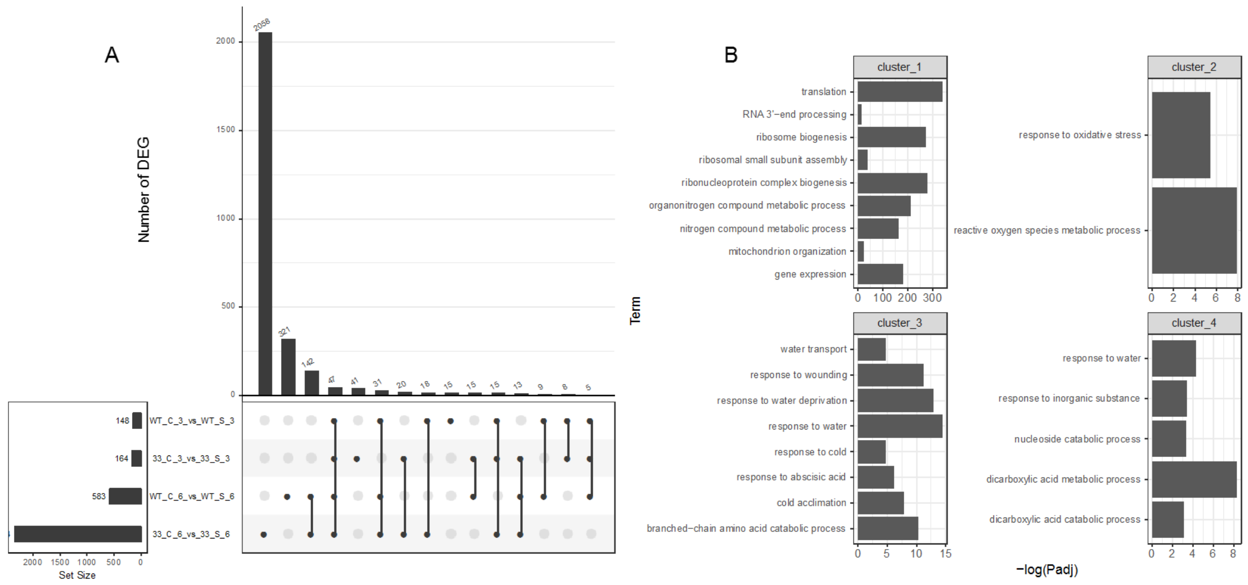

Figure S2: Genome-wide transcriptional effects of salt stress on the *rs33* mutant

(A) Overlap of differentially expressed genes (DEGs) between the *rs33* mutant and the WT. **(B)** Gene enrichment and clustering analysis of DEGs. Cluster 1 shows the enrichment of translation/ribosome biogenesis genes. Cluster 3 shows enrichment of stress-responsive genes.

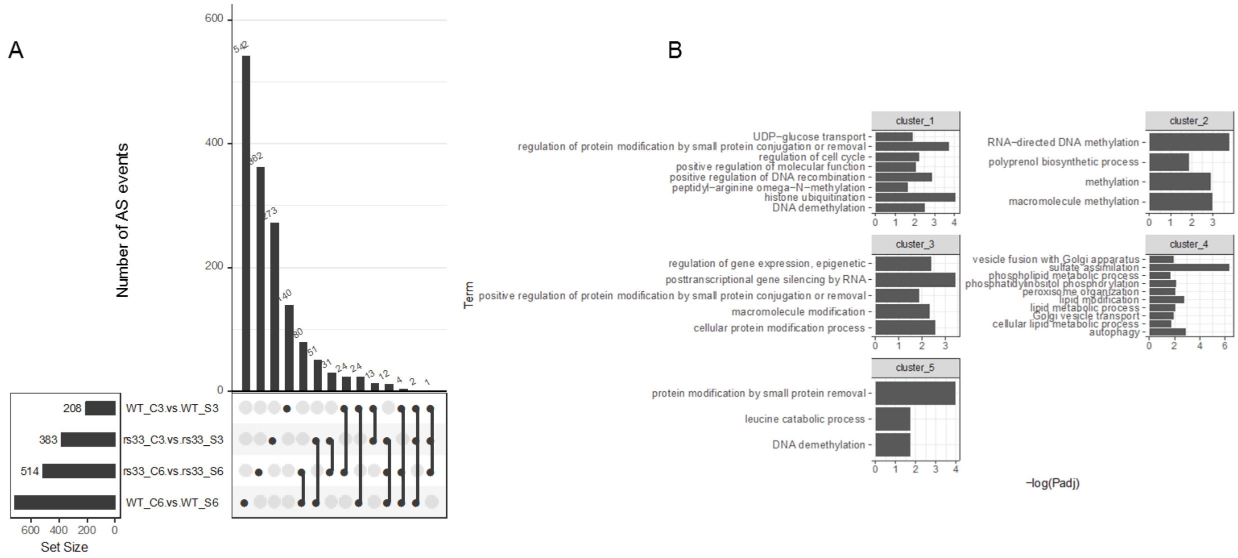

Figure S3: Impact of *OsRS33* on splicing efficiency under salt stress

(A) Upset plot showing the overlap of differential alternative splicing (DAS) events between *rs33* and WT plants under salt stress conditions. **(B)** Gene enrichment analysis of splicing change during the salt stress response in *rs33* and WT plants.

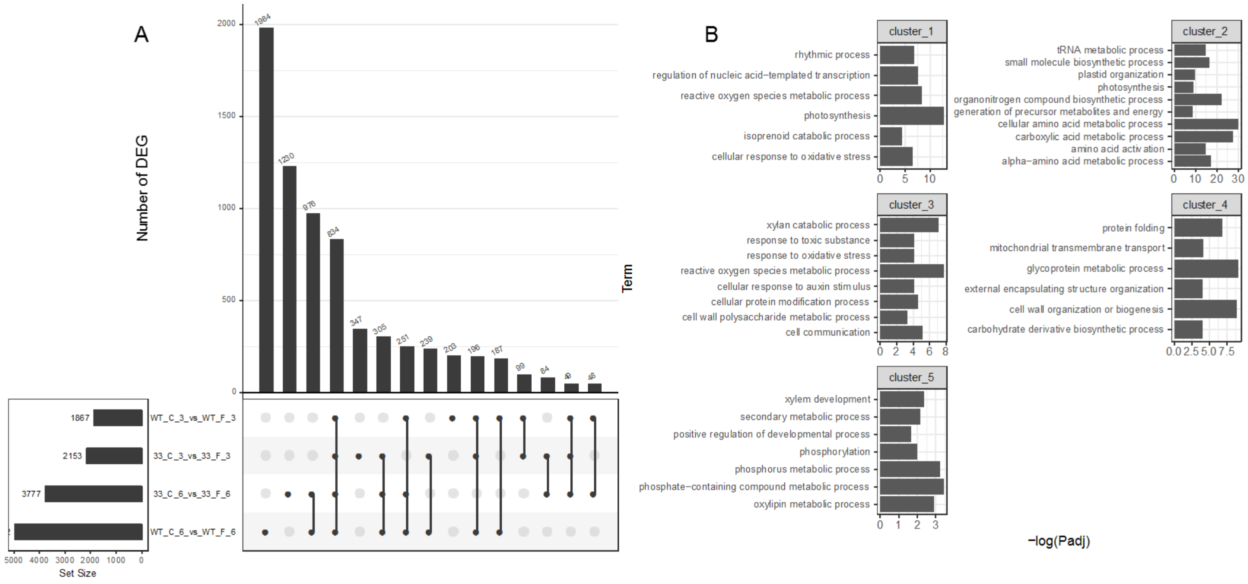

Figure S4: Effect of the *rs33* mutation on global gene expression under cold stress

(A) Overlap of differentially expressed genes (DEGs) between the *rs33* mutant and the WT. One-week-old rice seedlings were treated with cold stress (4ºC) for 3 and 6 hours and used for RNA extraction. **(B)** Gene enrichment analysis of the DEGs. Cluster 1 shows enrichment of oxidative stress response genes in *rs33* after 6 h of cold treatment as compared to the WT. Cluster 4 shows cell wall biogenesis genes enriched at 6 h of cold treatment in the *rs33* mutant.

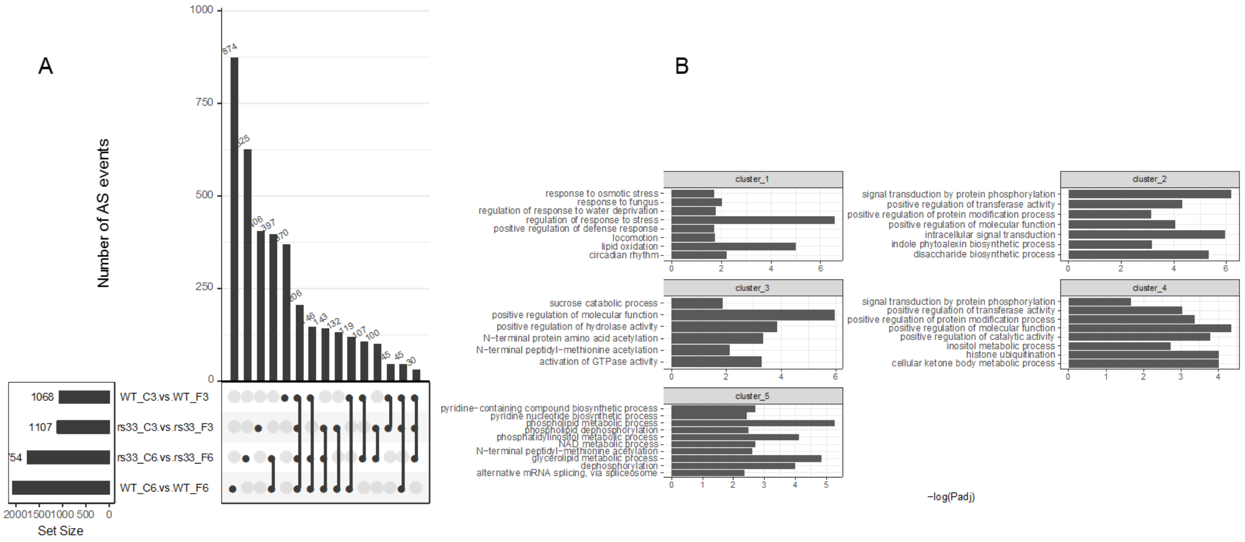

Figure S5: Comparison of global AS events between the WT and the rs33 mutant under cold stress

(A) Upset plot showing the overlap of differential alternative splicing (DAS) events between *rs33* and WT plants under cold stress conditions. **(B)** Gene enrichment analysis of splicing change during the salt stress response in *rs33* and WT plants. Cluster 1 contains a lot of stress-related genes with a quick AS regulation upon stress. Cluster 5 shows that some genes of the lipid metabolism pathway did not respond to cold in the *rs33* mutant.

Table S1: List of primer sequences used in this study

Locus	Name of the primer	Sequence (5' - 3')	Purpose
LOC_Os02g03040	RS33_F	CCTAGCATTGGCTATGGTGG	Genotyping of RS33
	RS33_R	CAAATGGAGTACCCACTCGC	Genotyping of RS33
LOC_Os04g02870	RS29_F	TCGTGGGGAACCTCGACTACG	Semi-quantitative RT-PCR
	RS29_R	TGCTTTTGTGGCCTCTTCCTG	Semi-quantitative RT-PCR
LOC_Os03g42820	LDCP_F	GACCCGGGTCTGGATTTTGTGG	Semi-quantitative RT-PCR
	LDCP_R	CCCCTTCCGCTGATCTTCTTCTG	Semi-quantitative RT-PCR
LOC_Os01g48960	GLT1_F	TAGCGGAATCGCTGGGACATG	Semi-quantitative RT-PCR
	GLT1_R	CAATTCTTGTTCCCTTTGAGGGTATTGATC	Semi-quantitative RT-PCR
LOC_Os08g05750	750PPR_F	CTGCTCCTGGGATGACCTTGAG	Semi-quantitative RT-PCR
	750PPR_R	ACCTCACTGTTATTCCTTTCCATGACC	Semi-quantitative RT-PCR
LOC_Os01g21420	SF2_F	CTCTGATGTATACCGTGAGGCTG	Semi-quantitative RT-PCR
	SF2_R	GGAGAGGGAGAGCGGGATAAG	Semi-quantitative RT-PCR
LOC_Os01g72370	bHLH56_F	GAAGTGAAGGAACGATCAACGAGG	Semi-quantitative RT-PCR
	bHLH56_R	CGGCATTACGCGCTGCTGATG	Semi-quantitative RT-PCR
LOC_Os01g57490	SDRLP_F	AAAGGGGGTGGACACTGACAG	Semi-quantitative RT-PCR
	SDRLP_R	CTTGGATGCAGCATCCCAAGAG	Semi-quantitative RT-PCR
LOC_Os04g57190	UCH5_F	ATGAGCTTGATGGTATGAAGCCTGG	Semi-quantitative RT-PCR
	UCH5_R	ACCAATAGTCATGTTGGATCAGTCG	Semi-quantitative RT-PCR
LOC_Os01g14440	WRKY1_F	CGCCGATGGGTGCCAATGGAG	Semi-quantitative RT-PCR
	WRKY1_R	GACCCCGACAGAAGCATCGACG	Semi-quantitative RT-PCR
LOC_Os02g14910	BZIP19_F	ATGTTCTCCGACGTCGAGGCG	Semi-quantitative RT-PCR
	BZIP19_R	CGACCGCTGATGATCAAGGAAG	Semi-quantitative RT-PCR
LOC_Os03g17760	760RBD_F	ACGCCACACCGGTCATCAACG	Semi-quantitative RT-PCR
	760RBD_F	GGGGAGAACGCCGTGGTATTG	Semi-quantitative RT-PCR
LOC_Os02g50140	140ABA_F	ATGGAGGTTCGTCGACGATGG	Semi-quantitative RT-PCR
	140ABA_R	GCCATCGGGTACAGTTTTTGCATG	Semi-quantitative RT-PCR
	RS33_sgRNA	AACAAAGCACCAGTGGTCTAGTGGTAGAATAGTACCCTGCCACGGTACAGACCCGGGTTCGATTCCCGGCTGGTGCAGAGCGCCTCTTCAGCAAATAGTTTTAGAGCTAGAAATAGCAAGTTAAAATAAGGCTAGTCCGTTATCAACTTGAAAAAGTGGCACCGAGTCGGTGCTTTTTTTTTT<br><br>pre-tRNA: yellow backgroundgRNA spacer: gray backgroundScaffold RNA: green backgroundpolIII terminator: red background	sgRNA synthetic fragment cloned into pRGEB32 via BsaI to target RS33 locus

Table S2: List of DEGs of all comparisons

Table S3: List of DAS of all comparisons
